# Risk of Type 2 Diabetes and Obesity Is Differentially Associated with Variation in *FTO* in Whites and African-Americans in the ARIC Study

**DOI:** 10.1371/journal.pone.0010521

**Published:** 2010-05-20

**Authors:** Jan Bressler, W. H. Linda Kao, James S. Pankow, Eric Boerwinkle

**Affiliations:** 1 Human Genetics Center, University of Texas Health Science Center at Houston, Houston, Texas, United States of America; 2 Department of Epidemiology, Johns Hopkins Bloomberg School of Public Health, Baltimore, Maryland, United States of America; 3 Division of Epidemiology and Community Health, School of Public Health, University of Minnesota, Minneapolis, Minnesota, United States of America; 4 Brown Foundation Institute of Molecular Medicine, University of Texas Health Science Center at Houston, Houston, Texas, United States of America; Mayo Clinic College of Medicine, United States of America

## Abstract

Single nucleotide polymorphisms (SNPs) in the fat mass and obesity associated (*FTO*) gene are associated with body mass index (BMI) in populations of European descent. The *FTO* rs9939609 variant, first detected in a genome-wide association study of diabetes, conferred an increased disease risk that was abolished after adjustment for BMI, suggesting that the association may be due to variation in adiposity. The relationship between diabetes, four previously identified *FTO* polymorphisms that span a 19.6-kb genomic region, and obesity was therefore evaluated in the biracial population-based Atherosclerosis Risk in Communities Study with the goal of further refining the association by comparing results between the two ethnic groups. The prevalence of diabetes and obesity (BMI ≥30 kg/m^2^) was established at baseline, and diabetes was determined by either self-report, a fasting glucose level ≥126 mg/dL, or non-fasting glucose ≥200 mg/dL. There were 1,004 diabetes cases and 10,038 non-cases in whites, and 670 cases and 2,780 non-cases in African-Americans. Differences in mean BMI were assessed by a general linear model, and multivariable logistic regression was used to predict the risk of diabetes and obesity. For white participants, the *FTO* rs9939609 A allele was associated with an increased risk of diabetes (odds ratio (OR)  = 1.19, p<0.001) and obesity (OR = 1.22, p<0.001) under an additive genetic model that was similar for all of the SNPs analyzed. In African-Americans, only the rs1421085 C allele was a determinant of obesity risk (OR = 1.17, p = 0.05), but was found to be protective against diabetes (OR = 0.79, p = 0.03). Adjustment for BMI did not eliminate any of the observed associations with diabetes. Significant statistical interaction between race and the *FTO* variants suggests that the effect on diabetes susceptibility may be context dependent.

## Introduction

Diabetes is an important risk factor for cardiovascular disease [1]. Obesity is an independent risk factor for diabetes [2] and either BMI or waist-to-hip ratio (WHR) has been commonly used as a surrogate measure of adiposity. From a public health perspective, obesity is an accessible target for intervention programs designed to reduce the incidence of diabetes [3].

The fat mass and obesity associated (*FTO)* gene maps to chromosome 16q12.2 and includes nine predicted exons (Genbank NM 00108432). Genetic variants in *FTO* have been reported to be associated with BMI in study populations of European descent [4], [5], [6], [7], [8], although functional polymorphisms in this gene have not yet been identified. Frayling et al. identified the *FTO* rs9939609 SNP in a genome-wide association study comparing 1,924 British type 2 diabetes cases with 2,938 controls [7]. The minor A allele was found to be strongly associated with diabetes, while adjustment for BMI in a replication sample abolished the association with diabetes. *FTO* is ubiquitously expressed in human tissues [6], [7], with a high level found in the hypothalamus indicating a possible role in the regulation of appetite or energy balance. In accordance with this hypothesis, introduction of a *Fto* null mutation in mice resulted in postnatal growth retardation, loss of white adipose tissue, and increased energy expenditure [9]. The ability of murine Fto to catalyze the demethylation of 3-methylthymine in single-stranded DNA has also recently been reported [10]. Although a relationship between *FTO* polymorphisms and obesity has been found in African-American children [11], there was no association between *FTO* variants and either diabetes or obesity in a study of postmenopausal women [12], and there have been inconsistent results for African-American adults in three other family-based studies [8], [13], [14]. To date there have been no investigations of the association between *FTO* variants and diabetes or obesity in a community-based study that included both African-American men and women.

In this study, the relationship between diabetes, four previously reported *FTO* SNPs [6], [7], [15], and obesity was examined in African-American and white individuals enrolled in the large prospective Atherosclerosis Risk in Communities (ARIC) study [16]. Only one of the genetic variants (rs1421085) was associated with both diabetes and obesity in African-Americans, while all of the *FTO* polymorphisms were determinants of disease risk in whites as shown in other studies [4], [5], [6], [7], [8], [15]. This may reflect differences in linkage disequilibrium (LD) between the genetic variants in populations of European and African ancestry [17], as well as the effects of other as yet uncharacterized gene-gene or gene-environment interactions that may be distinctive for each racial group.

## Results

A description of the study sample stratified by diabetes status and race is shown in Tables 1 and 2. The values for all clinical characteristics differed significantly between cases and non-cases for both whites and African-Americans, except there was a significantly greater frequency of males in the group of white diabetes cases when compared to those without diabetes while there was no relationship between gender and case status for African-Americans.

**Table 1:** Clinical characteristics and *FTO* SNP genotype frequencies in African-Americans stratified by diabetes case status.

Characteristics	Diabetes (n = 670)	Non-Cases (n = 2,780)	p
Age, years	55.3 (5.6)^a^	53.1 (5.8)^a^	<0.001
Male (%)	35.7	38.7	0.147*
European ancestry (%)	16.7	18.1	0.002
BMI (kg/m^2^)	32.0 (6.1)^a^	29.0 (6.0)^a^	<0.001
DBP, mm Hg	78.3 (12.0)^a^	80.0 (12.2)^a^	<0.001
SBP, mm Hg	133.3 (22.9)^a^	127.7 (20.8)^a^	<0.001
HDL, mg/dL	48.8 (15.0)^a^	56.3 (17.8)^a^	<0.001
LDL, mg/dL	143.6 (45.0)^a^	135.9 (42.0)^a^	<0.001
Glucose, mg/dL	199.7 (89.9)^a^	98.7 (10.1)^a^	<0.001
Insulin, pmol/L	342.8 (632.4)^a^	94.8 (69.9)^a^	<0.001

*FTO* SNP	Genotype/Allele	n	%	n	%	p
rs9939609	TT	197	30.1	734	27.3	0.364 *
	AT	310	47.3	1,309	48.8	
	AA	48	22.6	642	23.9	
	T	704	53.7	2,777	51.7	
	A	606	46.3	2,593	48.3	
rs17817449	TT	258	39.5	996	36.9	0.413*
	GT	299	45.8	1,270	47.0	
	GG	96	14.7	434	16.1	
	T	815	62.4	3,262	60.4	
	G	491	37.6	2,138	39.6	
rs805136	CC	225	34.2	861	31.6	0.403*
	AC	306	46.6	1,334	48.9	
	AA	126	19.2	533	19.5	
	C	756	57.5	3,056	56.0	
	A	558	42.5	2,400	44.0	
rs1421085	TT	551	83.9	2,153	79.0	0.014*
	CT	101	15.4	531	19.5	
	CC	5	0.8	41	1.5	
	T	1,203	91.6	4,837	88.8	
	C	111	8.4	613	11.2	

n, number; p, p-value, significance of difference between group means determined by t-test; BMI, body mass index; DBP, diastolic blood pressure; SBP, systolic blood pressure; HDL, high density lipoprotein; LDL, low density lipoprotein;

a mean and standard deviation; SNP, single nucleotide polymorphism;

*p-value Pearson chi-squared.

**Table 2:** Clinical characteristics and *FTO* SNP genotype frequencies in whites stratified by diabetes case status.

Characteristics	Diabetes (n = 1,004)	Non-Cases (n = 10,038)	p
Age, years	56.2 (5.6)^a^	54.2 (5.7)^a^	<0.001
Male (%)	53.0	46.6	<0.001*
BMI (kg/m^2^)	30.4 (5.7)^a^	26.7 (4.6)^a^	<0.001
DBP, mm Hg	72.9 (10.9)^a^	71.4 (10.0)^a^	<0.001
SBP, mm Hg	126.8 (18.4)^a^	117.6 (16.6)^a^	<0.001
HDL, mg/dL	41.4 (13.9)^a^	51.4 (16.8)^a^	<0.001
LDL, mg/dL	140.1 (39.2)^a^	137.3 (37.7)^a^	0.032
Glucose, mg/dL	174.7 (70.6)^a^	98.6 (9.0)^a^	<0.001
Insulin, pmol/L	222.7 (433.6)^a^	72.6 (54.3)^a^	<0.001

*FTO* SNP	Genotype/Allele	n	%	n	%	p
rs9939609	TT	314	31.8	3,566	36.0	0.001*
	AT	472	47.8	4,739	47.8	
	AA	202	20.4	1,610	16.2	
	T	1,100	55.7	11,871	59.9	
	A	876	44.3	7.959	40.1	
rs17817449	TT	312	31.6	3,565	35.8	0.002*
	GT	474	48.1	4,752	47.8	
	GG	200	20.3	1.631	16.4	
	T	1,098	55.7	11,882	59.7	
	G	874	44.3	8,014	40.3	
rs805136	CC	311	31.6	3,545	35.9	0.001*
	AC	473	48.1	4,714	47.8	
	AA	200	20.3	1,614	16.3	
	C	1,095	55.6	11,804	59.8	
	A	873	44.4	7,942	40.2	
rs1421085	TT	304	30.8	3,461	35.0	0.001*
	CT	477	48.2	4,759	48.1	
	CC	208	21.0	1,673	16.9	
	T	1,085	54.8	11,681	59.0	
	C	893	45.2	8,105	41.0	

n, number; p, p-value, significance of difference between group means determined by t-test; BMI, body mass index; DBP, diastolic blood pressure; SBP, systolic blood pressure; HDL, high density lipoprotein; LDL, low density lipoprotein;

a mean and standard deviation; SNP, single nucleotide polymorphism;

*p-value Pearson chi-squared.

The allele and genotype frequencies for all of the *FTO* polymorphisms were in accordance with Hardy-Weinberg equilibrium expectations for both African-American and white individuals (p<0.05). There was a significant difference in the four *FTO* genotype frequencies between diabetes cases and non-cases found for whites, but a difference was only revealed for rs1421085 in African-Americans with the frequency of the minor allele higher in non-cases than in cases. Similarly, when the frequency of all possible two-, three-, and four-marker haplotypes was compared between diabetes cases and non-cases, significant differences were shown for all of the haplotypes in whites but for none of the marker combinations in African-Americans (Table S1).

A general linear model was used to determine whether there was significant variation in mean BMI between those with different *FTO* genotypes (Table 3). There was a significant difference found for white participants for all polymorphisms studied but only for rs17817449 and rs1421085 in African-Americans, although the trend in both racial groups was for increasing BMI with each addition of an *FTO* risk allele. There was also a significant difference found in mean WHR and waist circumference (WC) for white but not for African-American individuals categorized by *FTO* genotype, while there was no apparent variation in mean height with genotype for either racial group. Sex-specific analyses were carried out (data not shown) and variation in the effect of the *FTO* SNPs on obesity-related quantitative traits by gender was not observed with the exception of higher mean BMI found for African-American females (p = 0.02) but not for males (p = 0.55) with addition of a rs1421085 C allele.

**Table 3:** Association of *FTO* genotype and quantitative traits stratified by race.

SNP	Genotype	BMI (AA)	p*	WHR (AA)	p*	Waist (AA)	p*	Height (AA)	p*
rs9939609	TT	29.4 (6.2)^a^	0.22	0.918 (0.076)	0.82	98.7 (15.2)	0.27	167.9 (9.2)	0.63
	AT	29.6 (6.2)		0.919 (0.075)		99.1 (15.3)		168.2 (8.9)	
	AA	29.8 (5.9)		0.919 (0.076)		99.5 (14.8)		168.2 (8.8)	
rs17817449	TT	29.4 (6.1)	0.05	0.918 (0.075)	0.75	98.6 (14.9)	0.07	167.9 (9.0)	0.38
	GT	29.5 (6.1)		0.918 (0.077)		98.9 (15.1)		168.2 (8.9)	
	GG	30.0 (6.2)		0.920 (0.073)		100.1 (15.6)		168.4 (8.8)	
rs8050136	CC	29.6 (6.2)	0.48	0.919 (0.076)	0.77	99.1 (15.2)	0.58	167.8 (8.9)	0.59
	AC	29.5 (6.2)		0.918 (0.076)		98.8 (15.2)		168.2 (8.9)	
	AA	29.8 (6.0)		0.919 (0.075)		99.6 (15.0)		168.2 (8.8)	
rs1421085	TT	29.5 (6.1)	0.02	0.918 (0.076)	0.39	99.0 (15.0)	0.09	168.0 (8.9)	0.48
	CT	29.7 (6.3)		0.919 (0.074)		99.1 (15.3)		168.1 (9.0)	
	CC	30.3 (6.7)		0.926 (0.074)		101.4 (16.0)		170.1 (9.0)	

		BMI (W)	p*	WHR (W)	p*	Waist (W)	p*	Height (W)	p*
rs9939609	TT	26.6 (4.6)	<0.001	0.924 (0.079)	<0.001	95.2 (12.9)	<0.001	168.7 (9.4)	0.67
	AT	27.1 (4.9)		0.929 (0.079)		96.4 (13.4)		168.5 (9.4)	
	AA	27.5 (5.1)		0.935 (0.078)		97.7 (13.9)		168.8 (9.4)	
rs17817449	TT	26.6 (4.6)	<0.001	0.924 (0.080)	<0.001	95.2 (13.0)	<0.001	168.7 (9.4)	0.89
	GT	27.1 (4.9)		0.929 (0.079)		96.4 (13.5)		168.5 (9.4)	
	GG	27.5 (5.1)		0.935 (0.078)		97.6 (13.9)		168.8 (9.4)	
rs8050136	CC	26.6 (4.6)	<0.001	0.924 (0.080)	<0.001	95.2 (13.0)	<0.001	168.7 (9.4)	0.79
	AC	27.1 (4.9)		0.929 (0.079)		96.4 (13.5)		168.5 (9.4)	
	AA	27.5 (5.1)		0.935 (0.078)		97.7 (13.9)		168.8 (9.4)	
rs1421085	TT	26.6 (4.6)	<0.001	0.924 (0.79)	<0.001	95.1 (13.0)	<0.001	168.7 (9.4)	0.75
	CT	27.1 (4.9)		0.924 (0.79)		96.5 (13.5)		168.5 (9.4)	
	CC	27.5 (5.1)		0.934 (0.078)		97.6 (13.9)		168.8 (9.4)	

SNP, single nucleotide polymorphism; BMI, body mass index; AA, African-American; p, p-value, significance of differences in means of obesity-related quantitative traits among individuals categorized by *FTO* genotype;

*adjusted for age and gender in white participants, and for age, gender, and percentage European ancestry in African-American participants; WHR, waist-to-hip ratio;

a mean and standard deviation; W, white.

BMI was also evaluated as a categorical variable using standard criteria [18] (Table 4). When a BMI ≥30 kg/m^2^ was used to define obesity and logistic regression models were adjusted for age and gender, addition of an *FTO* rs1421085 C allele conferred an elevated risk of being obese for both white and African-American participants. The remaining 3 *FTO* polymorphisms were significantly associated with obesity in whites but not in African-Americans. If this association was examined separately by gender, the risk of obesity was similar for both sexes for white participants for all of the polymorphisms, while a significantly increased risk of obesity was found for African-American males but not for females for rs9939609 (males, OR = 1.27, p = 0.01; females, OR = 1.00, p = 0.95) and rs17817449 (males, OR = 1.19, p = 0.05; females, OR = 1.06, p = 0.36) with evidence for gender by gene interaction for rs9939609 (OR for interaction  = 1.27, p = 0.03). Application of a polytomous logistic model revealed a significant association between the 4 *FTO* genetic variants and BMI across all categories for white participants when compared to individuals whose BMI was <25 kg/m^2^, while this pattern was not observed for African-American subjects.

**Table 4:** Association of *FTO* and obesity or BMI stratified by race.

SNP	BMI	kg/m^2^	AA	%	OR	95% CI	p*	White	%	OR	95% CI	p*
rs9939609		Obese (≥30)	1,346	40.3	1.09	0.98–1.20	0.10	2,470	22.6	1.22	1.14–1.30	<0.001
		<25	730	21.9	†			4,065	37.3	†		
		25–29	1,264	37.8	1.01	0.89 –1.15	0.86	4,368	40.0	1.08	1.02–1.15	0.02
		30–40	1,154	34.5	1.10	0.96–1.26	0.15	2,276	20.9	1.27	1.18–1.37	<0.001
		>40	192	5.8	1.04	0.83–1.31	0.70	194	1.8	1.23	1.00 –1.51	0.05
rs17817449		Obese (≥30)	1,346	40.1	1.10	1.00–1.22	0.06	2,473	22.6	1.20	1.13–1.28	<0.001
		<25	741	22.1	†			4,087	37.4	†		
		25–29	1,266	37.8	0.98	0.86–1.11	0.73	4,374	40.0	1.09	1.02–1.16	0.01
		30–40	1,155	34.4	1.08	0.95–1.24	0.24	2,276	20.8	1.26	1.17–1.36	<0.001
		>40	191	5.7	1.09	0.86–1.38	0.47	197	1.8	1.26	1.03–1.55	0.02
rs8050136		Obese (≥30)	1,362	40.2	1.04	0.94–1.15	0.42	2,470	22.8	1.21	1.13–1.29	<0.001
		<25	741	21.9	†			4,050	37.3	†		
		25–29	1,282	37.9	1.02	0.89 –1.16	0.80	4,337	40.0	1.10	1.03–1.17	0.01
		30–40	1,167	34.5	1.06	0.93–1.21	0.40	2,275	20.9	1.27	1.18–1.37	<0.001
		>40	195	5.7	1.00	0.81–1.28	0.90	195	1.8	1.25	1.02–1.54	0.03
rs1421085		Obese (≥30)	1,358	40.2	1.17	1.00–1.38	0.05	2,471	22.7	1.22	1.15–1.30	<0.001
		<25	739	21.9	†			4,059	37.3	†		
		25–29	1,285	38.0	1.02	0.83–1.26	0.83	4,352	40.0	1.10	1.03–1.17	0.01
		30–40	1,164	34.4	1.18	0.95 –1.47	0.13	2,273	20.9	1.28	1.19–1.38	<0.001
		>40	194	5.7	1.25	0.86 –1.81	0.24	198	1.8	1.26	1.03–1.54	0.03

SNP, single nucleotide polymorphism; BMI, body mass index; AA, African-American; OR, odds ratio; CI, confidence interval; p, p-value for multivariable logistic regression (additive genetic model) used to predict risk of obesity (BMI ≥30 kg/m^2^), or for polytomous logistic regression used across categories of BMI with BMI <25 kg/m^2^ as the common reference;

*adjusted for age and gender in white participants, and for age, gender, and percentage European ancestry in African-American participants;

†, referent group.

Multivariable logistic regression models were used to examine the association of sequence variation in the *FTO* gene and diabetes case status. These results are presented in Table 5. After adjusting for age and gender, all of the *FTO* risk alleles were significantly associated with an increased susceptibility for diabetes in white individuals. Adjustment for age, gender, as well as BMI attenuated but did not abolish the risk for diabetes.

**Table 5:** Association of *FTO* risk alleles and diabetes stratified by race.

*FTO* SNP	Race	Subjects (n)	Diabetes (n)	%	OR^1^	95% CI	p	OR^2^	95% CI	p
rs9939609	AA	3,340	655	19.6	0.92	0.82–1.04	0.18	0.91	0.80–1.03	0.12
	White	10,903	988	9.1	1.19	1.09–1.31	<0.001	1.12	1.02–1.23	0.02
rs17817449	AA	3,353	653	19.5	0.92	0.81–1.04	0.17	0.89	0.79 –1.02	0.08
	White	10,934	986	9.0	1.18	1.08–1.30	<0.001	1.11	1.01–1.22	0.03
rs8050136	AA	3,385	657	19.4	0.94	0.83–1.06	0.34	0.94	0.83–1.06	0.29
	White	10,882	984	9.1	1.19	1.08–1.31	<0.001	1.12	1.01–1.23	0.02
rs1421085	AA	3,382	657	19.4	0.79	0.64 –0.98	0.03	0.75	0.60 –0.93	0.01
	White	10,857	989	9.1	1.19	1.09–1.31	<0.001	1.12	1.01–1.23	0.02

SNP, single nucleotide polymorphism; n, number; OR^1^, odds ratio, adjusted for age and gender in white participants, and for age, gender, and percentage European ancestry in African-American participants; CI, confidence interval; OR^2^, odds ratio, adjusted for age, gender, and BMI in white participants, and for age, gender, BMI, and percentage European ancestry in African-American participants; p, p-value for multivariable logistic regression (additive genetic model); AA, African-American.

In contrast to the results described above for whites, three of the *FTO* SNPs were not significantly associated with diabetes case status for African-American participants in the ARIC study (Table 5). However, a significant protective effect of the rs142105 C allele was observed that was still evident after adjustment for BMI. When multivariable logistic regression was performed to examine the influence of the *FTO* gene on diabetes risk after combining African-American and white participants and adjusting for age, gender, and race, significant interaction between all of the *FTO* genetic variants and race was detected (p-value for interaction  = 0.001, 0.001, 0.002, and <0.001 for rs9939609, rs17817449, rs805136, and rs1421085, respectively).

## Discussion

The relationship between diabetes, four previously identified *FTO* polymorphisms, and obesity was evaluated in the biracial population-based ARIC cohort study in an initial effort to fine map the association by comparing results between two racial groups. The assumption underlying the performance of these analyses was that since functional SNPs in *FTO* have not yet been identified, the detection of genetic variants that are significantly associated with obesity and diabetes in multiple ethnic or racial groups may help to further define the relevant genomic region or potential candidate causative mutations. In this study, all of the *FTO* polymorphisms were associated with elevated risk for diabetes and obesity in white participants, while addition of the rs1421085 C allele was a determinant of increased susceptibility to obesity in African-Americans but conferred a protective effect against diabetes.

Although Frayling et al. found that the association of the *FTO* gene and diabetes was mediated by BMI in a genome-wide association study of British diabetes cases and controls [7], the results for this cohort suggest that the gene plays a role in the susceptibility to diabetes that cannot entirely be accounted for by its effect on body size. The attenuation but not elimination of risk for diabetes after adjusting for BMI in whites may reflect the fact that a single BMI measurement in prevalent cases may not fully capture the effect of *FTO* on adiposity and metabolism. The protective effect of the rs1421085 C allele was more marked after adjustment for BMI in African-Americans. For both racial groups, the effect sizes prior to adjustment were lower than that reported by Frayling et al. [7] for the Wellcome Trust Case Control Consortium but were similar to the results found for participants in the Finland-United States Investigation of the Non-Insulin-Dependent Diabetes Mellitus Genetics (FUSION) study in another early genome-wide association analysis of diabetes risk [15], [19].

Genetic variants in the *FTO* gene have consistently been reported to be associated with BMI [4], [5], [6], [7], [8] and diabetes [7], [15] in Europeans, but the results have been variable for replication in other ethnicities including Hispanics [8], [12], [14], Asian and Oceanic populations [12], [20], [21], [22], [23], [24], [25], [26], [27], [28], [29], [30], [31], and African-Americans [8], [11], [12], [13], [14]. In a case-control study of *FTO* and childhood obesity defined as a BMI ≥95^th^ percentile, only rs3751812 was shown to confer significant risk in African-American members of the cohort, while 7 out of the 13 SNPs genotyped were significantly associated with the same trait in Caucasians. As in the case of rs1421085 in the current study (Tables 1, 2, and S2), rs3751812 is not in strong LD in HapMap African ancestry populations with the *FTO* rs9939609 variant first identified in Europeans (r^2^ = 0.072 in Yoruba in Ibadan, Nigeria (YRI); r^2^ = 0.119 in African ancestry in southwest USA (ASW)), and the minor allele frequency (MAF) was not the same for white and African-American participants enrolled in the cohort (MAF for rs3751812  = 0.443 and 0.115 in Caucasian and African-Americans, respectively), indicating that this polymorphism may be associated differently with a putative causative variant in the two racial groups [11]. In contrast, when 27 *FTO* variants were genotyped in 604 African-American subjects enrolled in the Insulin Resistance Atherosclerosis Family Study, both rs9939609 and rs8050136 were significantly associated with BMI and WC, but no association was observed for either rs3751812 or rs1421085 [14]. Scuteri et al. carried out a genome-wide association scan in 6,148 individuals from a genetically isolated population in Sardinia and identified 8 SNPs within the *FTO* gene that were associated with increased BMI, hip circumference, and weight. When either the SNP that was most highly associated with all 3 quantitative traits in Sardinia (rs9930506) or 7 additional SNPs were genotyped in 1,101 African-American participants in the GenNet study, there was no evidence for association with BMI. The authors suggested that because the MAF for some of the *FTO* variants tested, including rs1421085, was lower in African-Americans than in European Americans, increased sample sizes would be required to assess their effects [8]. A similar difference in allele frequency was observed in the study reported here (MAF for rs1421085  = 0.41 and 0.11 in whites and African-Americans, respectively) while the study population examined was considerably larger (n = 3,450 African-Americans), possibly enabling the detection of an association between this variant and an obesity-related quantitative trait in the ARIC cohort. There was also no association observed between 2 *FTO* polymorphisms (rs9939609 and rs8050136) and obesity, BMI, WC, or diabetes in African-American participants in a nested case-control study of postmenopausal women from the Women's Health Initiative-Observational Study [12], or between *FTO* variants and either BMI or weight in a genome-wide association study that included a group of 1,160 African-Americans in the discovery sample [13].

Evidence of statistical interaction between race and the *FTO* polymorphism shown after combining African-American and white participants further suggests that the influence of the *FTO* gene on diabetes susceptibility may be context dependent. There are a number of potential explanations for the difference in the impact of this gene on disease risk in white and African-American participants in the ARIC study where there was adequate power to detect an effect of the same magnitude (OR = 1.15–1.27) previously reported for Europeans [7], [15], [19] (Table S3). One factor may be the degree of LD between the polymorphisms since all of the *FTO* SNPs are highly correlated (r^2^>0.90) in whites of European descent (CEU) whereas the strength of LD across the 19.6-kb region within intron 1 is reduced in populations with African ancestry included in the International HapMap project [17] (Table S2). The contribution of all four of the SNPs to increased diabetes risk in whites is in accordance with the LD pattern in the region so that the analysis of each of the variants would be expected to yield a similar measure of association. In addition, the differences in LD among the SNPs when whites are compared to African-Americans could lead to the observed results if the true causative mutation is in turn correlated with the four *FTO* variants examined in whites but only with rs1421085 in African-Americans. Another possibility is that if the relationship between *FTO* sequence variation and diabetes is at least partially mediated by BMI, the nominally significant p-value found for the association of rs1421085 with obesity and the absence of association for the remaining variants in African-Americans may make an increase in diabetes susceptibility more difficult to detect. Alternatively, although it is necessary to treat this speculation with considerable caution, the genetic architecture of diabetes may differ in the two ethnic groups. Detection of a protective effect of the rs1421085 C allele against diabetes in African-Americans while risk is increased in whites, even though the same allele marginally increases susceptibility to obesity in both racial groups, provides some support for this view. Finally, variation in other genetic or environmental factors that contribute to the development of type 2 diabetes may underlie the apparently disparate effects of the *FTO* gene in African-Americans and whites in the ARIC cohort. More extensive genotyping of common polymorphisms in other populations combined with genomic sequencing strategies to catalog the contribution of rare variants will be required to confirm the observed racial differences in the effect of the *FTO* SNPs on diabetes susceptibility, and may help to distinguish between these various possibilities.

## Materials and Methods

### Ethics Statement

All individuals enrolled in the ARIC study provided written informed consent, and the study design and methods were approved by institutional review boards at the four collaborating medical centers: University of Mississippi Medical Center Institutional Review Board (Jackson Field Center); Wake Forest University Health Sciences Institutional Review Board (Forsyth County Field Center); University of Minnesota Institutional Review Board (Minnesota Field Center); and the Johns Hopkins School of Public Health Institutional Review Board (Washington County Field Center).

### Atherosclerosis Risk in Communities (ARIC) Study

The ARIC Study is a prospective longitudinal investigation of the development of atherosclerosis and its clinical sequelae involving 15,792 individuals aged 45 to 64 years at baseline selected by probability sampling from four communities in the United States. A detailed description of the ARIC Study has been reported previously [16]. At the inception of the study in 1986–1989, the participants were recruited from Forsyth County, North Carolina; Jackson, Mississippi (African-Americans only); the northwestern suburbs of Minneapolis, Minnesota; or Washington County, Maryland. Four examinations have been carried out at three-year intervals (exam 1, 1987–1989; exam 2, 1990–1992; exam 3, 1993–1995; exam 4, 1996–1998), and subjects are contacted annually to update their medical histories between examinations. Individuals were excluded from this analysis if they restricted use of their DNA (n = 44), were African-Americans from the Minnesota or Maryland field centers because of the small numbers of individuals recruited from these sites (n = 55), were neither African-American nor white (n = 48), or were missing all *FTO* genotypes (n = 599), diabetes case status (n = 102), BMI (n = 12), WC (n = 3), WHR (n = 4), or estimated percentage European ancestry (n = 433). The final study sample consisted of 14,492 participants.

### Clinical and Laboratory Measurements at the Baseline Examination

The prevalence of diabetes was determined at the baseline examination using a fasting glucose level ≥126 mg/dl, a nonfasting glucose level ≥200 mg/dl, and/or self-reported physician diagnosis or treatment for diabetes. Body weight and other anthropometric variables were measured by trained technicians according to standardized protocols. BMI was calculated as weight in kilograms/(height in meters)^2^ and obesity was defined as BMI ≥30. WHR was calculated as waist girth in cm/hip girth in cm. Blood pressure was measured three times while seated using a random-zero sphygmomanometer and the last two measurements were averaged for analysis. Plasma total cholesterol was measured by an enzymatic method [32] and low density lipoprotein (LDL) cholesterol was calculated [33]. High density lipoprotein (HDL) cholesterol was measured after dextran-magnesium precipitation of non-HDL [34]. Fasting serum glucose was measured by a standard hexokinase method on a Coulter DACOS chemistry analyzer (Coulter Instruments, Fullerton, CA, USA) and the fasting serum insulin level was assessed by radioimmunoassay (^125^Insulin Kit, Cambridge Medical Diagnostics, Billerica, MA, USA).

### Genotype Determination

Genotyping of the *FTO* polymorphisms was performed using the TaqMan assay (Applied Biosystems, Foster City, CA, USA). Sequences for primers and TaqMan probes are available upon request. Allele detection was performed using the ABI Prism 7700 Sequence Detection System (Applied Biosystems). The genotype call rate, or the percentage of samples to which a genotype was assigned, was determined prior to exclusion of individuals from the analysis and ranged from 94.4% for rs9939609 and rs8050136 to 94.7% for rs1781744. After the application of all exclusion criteria, the proportion of missing genotype data in the final study sample did not exceed 2.5% for any of the genetic variants. The genotyping success rate was also assessed by analyzing the concordance between genotypes for pairs of blind duplicates included with the DNA samples from the study participants. Kappa coefficients [35], an index of the percent agreement between measurements corrected for agreement occurring by chance, were calculated for each SNP and ranged from 0.97 to 0.98.

### Statistical Analysis

All statistical analyses were performed using Stata 9 software (StataCorp, College Station, TX, USA). Hardy-Weinberg equilibrium was tested using a χ^2^ goodness-of-fit test for all individuals in each racial group. The proportions, means and standard deviations were calculated for established risk factors for diabetes for both the prevalent diabetes cases and for the comparison groups. Multivariable logistic regression was used to evaluate the relationship between diabetes case status, the *FTO* alleles, and measures of obesity under an additive genetic model. Tests of interaction were conducted by including main effects for each *FTO* polymorphism and gender or race in the logistic regression models, and introducing a multiplicative two-way interaction term for gene by gender or gene by race. Prevalence odds ratios (OR) were adjusted for age and gender for white participants, and age, gender, and percentage European ancestry for African-American participants [36]. Percentage European ancestry was determined by performing a genome-wide admixture mapping scan using a panel of 1,350 ancestry informative markers and the Illumina BeadLab platform (Illumina, San Diego, CA, USA). Global ancestry was estimated using ANCESTRYMAP software [37]. General linear models were used to assess mean differences in BMI, WHR, and WC among *FTO* SNP genotypes. A p-value <0.05 was considered statistically significant. The results of all statistical analyses are reported separately by self-reported racial group.

## Supporting Information

Table S1 Haplotype analysis stratified by race.
(0.04 MB DOC)

Table S2 Linkage disequilibrium (LD) between *FTO* SNPs in HapMap populations.
(0.05 MB DOC)

Table S3 Prevalence odds ratios for diabetes and obesity case status detectable at 80% power stratified by race.
(0.03 MB DOC)
