# Supplementary material for: Risk of Type 2 Diabetes and Obesity Is Differentially Associated with Variation in FTO in Whites and African-Americans in the ARIC Study
Source: PLoS One. 2010 May 20;5(5):e10521. doi: 10.1371/journal.pone.0010521 (PMC2873943; doi:10.1371/journal.pone.0010521)
Supplement: Table S1 — Haplotype analysis stratified by race. (0.04 MB DOC) [file pone.0010521.s001.doc]

| **Haplotypes** | ***FTO* SNPs** | **Diab. (p^AA)** | **Diab. (p*W)** | **Obes. (p^AA)** | **Obes. (p*W)** |
| --- | --- | --- | --- | --- | --- |
|  |  |  |  |  |  |
| 2-marker |  |  |  |  |  |
|  | rs1421085_rs178171449 | 0.103 | 0.001 | 0.114 | <0.001 |
|  | rs178171449_rs8050136 | 0.244 | 0.001 | 0.071 | <0.001 |
|  | rs8050136_rs9939609 | 0.625 | 0.001 | 0.334 | <0.001 |
| 3-marker |  |  |  |  |  |
|  | rs1421085_rs17817449_rs8050136 | 0.175 | 0.001 | 0.076 | <0.001 |
|  | rs17817449_rs8050136_rs9939609 | 0.581 | 0.001 | 0.098 | <0.001 |
| 4-marker |  |  |  |  |  |
|  | rs1421085_rs17817449_rs8050136_rs9939609 | 0.318 | 0.001 | 0.067 | <0.001 |

Table S1. **Haplotype analysis stratified by race**. Haplotype association analysis was performed using

a moving window approach and Helix Tree software (Golden Helix Inc., Bozeman, MT). Haplotype

frequencies were inferred using the expectation maximization (EM) algorithm. SNP, single nucleotide

polymorphism; Diab., diabetes case status; p, p-value, p-value Pearson chi-squared for comparison of

frequency of all possible haplotypes between cases and non-cases; ^adjusted for age, gender, and

percentage European ancestry; AA, African-American; *adjusted for age and gender; Obes., obesity

case status
