# Supplementary material for: Risk of Type 2 Diabetes and Obesity Is Differentially Associated with Variation in FTO in Whites and African-Americans in the ARIC Study
Source: PLoS One. 2010 May 20;5(5):e10521. doi: 10.1371/journal.pone.0010521 (PMC2873943; doi:10.1371/journal.pone.0010521)
Supplement: Table S2 — Linkage disequilibrium (LD) between FTO SNPs in HapMap populations. (0.05 MB DOC) [file pone.0010521.s002.doc]

| **LD** | ***FTO* SNP** | **rs9939609** | **rs17817449** | **rs8050136** | **rs1421085** |
| --- | --- | --- | --- | --- | --- |
| r^2^, CEPH |  |  |  |  |  |
|  | rs9939609 | x | 1.00 | 1.00 | 0.936 |
|  | rs17817449 | 1.00 | x | 1.00 | 0.936 |
|  | rs8050136 | 1.00 | 1.00 | x | 0.936 |
|  | rs1421085 | 0.936 | 0.936 | 0.936 | x |
| r^2^, YRI |  | rs9939609 | rs17817449 | rs8050136 | rs1421085 |
|  | rs9939609 | x | 0.63 | 0.828 | 0.072 |
|  | rs17817449 | 0.63 | x | 0.751 | 0.117 |
|  | rs8050136 | 0.828 | 0.751 | x | 0.087 |
|  | rs1421085 | 0.072 | 0.117 | 0.087 | x |
| r^2^, ASW |  | rs9939609 | rs17817449 | rs8050136 | rs1421085 |
|  | rs9939609 | x | 0.745 | 0.838 | 0.119 |
|  | rs17817449 | 0.745 | x | 0.889 | 0.159 |
|  | rs8050136 | 0.838 | 0.889 | x | 0.141 |
|  | rs1421085 | 0.119 | 0.159 | 0.141 | x |

Table S2. **Linkage disequilibrium (LD) between *FTO* SNPs in**

**HapMap populations.** SNP, single nucleotide polymorphism;

CEPH, Utah residents with northern and western European ancestry;

YRI, Yoruba in Ibadan, Nigeria; ASW, African ancestry in southwest

USA; LD data is for autosomes genotyped in HapMap phase 3

(Release 27, February 2009) [17]
