# Supplementary material for: Risk of Type 2 Diabetes and Obesity Is Differentially Associated with Variation in FTO in Whites and African-Americans in the ARIC Study
Source: PLoS One. 2010 May 20;5(5):e10521. doi: 10.1371/journal.pone.0010521 (PMC2873943; doi:10.1371/journal.pone.0010521)
Supplement: Table S3 — Prevalence odds ratios for diabetes and obesity case status detectable at 80% power stratified by race. (0.03 MB DOC) [file pone.0010521.s003.doc]

| ***FTO* SNP** | **OR (AA) Obesity** | **OR (AA) Diabetes** | **OR (W) Obesity** | **OR (W) Diabetes** |
| --- | --- | --- | --- | --- |
|  |  |  |  |  |
| rs9939609 | 1.150 | 1.189 | 1.097 | 1.144 |
| rs17817449 | 1.154 | 1.193 | 1.097 | 1.144 |
| rs8050136 | 1.150 | 1.189 | 1.097 | 1.144 |
| rs1421085 | 1.244 | 1.301 | 1.097 | 1.143 |

Table S3. **Prevalence odds ratios for diabetes and obesity case status**

**detectable at 80% power stratified by race.** Power analyses were conducted

with the program Quanto (<http://hydra.usc.edu/GxE/>) using a fixed sample size

for African-Americans and whites for each *FTO* polymorphism, diabetes and

obesity as categorical variables, a p-value of 0.05, and the allele frequencies in

the study sample. SNP, single nucleotide polymorphism; OR, odds ratio, logistic

regression (additive genetic model); AA, African-American; W, white
